# Spontaneous Unilateral Carotid Dissection: A Case Report of a Rare Complication in a Patient With Graves' Disease

**DOI:** 10.7759/cureus.72298

**Published:** 2024-10-24

**Authors:** Jorge Eduardo Contreras-Saldarriaga, Angela María López-Arbelaez, Alejandra Solano-Villamarin, Oriana Fiorella Arroyo-Ripoll, Alejandro Román-González

**Affiliations:** 1 Department of Endocrinology, University of Antioquia, Medellín, COL; 2 Department of Internal Medicine, University of Antioquia, Medellín, COL; 3 Department of Vascular Medicine, University of Antioquia, Medellin, COL; 4 Department of Endocrinology and Metabolism, University of Antioquia, Hospital San Vicente Foundation, Medellín, COL

**Keywords:** carotid artery dissection, dissection of cervical arteries, graves' disease, hyperthyroidism, stroke, thyroid autoimmunity, thyroid storm, thyrotoxicosis

## Abstract

Carotid artery dissection (CAD) is a recognized cause of ischemic stroke (IS) in young adults. At the same time, hyperthyroidism, particularly in the context of thyroid storm (TS), can also lead to IS through mechanisms related and unrelated to atrial fibrillation (AF). However, the coexistence of CAD and thyrotoxicosis is extremely rare. We report the case of a 45-year-old woman with Graves' disease (GD) who presented with TS, developing IS secondary to left CAD. The patient had a history of poorly controlled hyperthyroidism despite being on methimazole and beta-blocker therapy. Upon admission, she presented with fever, diarrhea, tremor, and palpitations. Physical examination revealed bilateral exophthalmos, goiter, and AF. Thyroid function tests confirmed TS, and treatment was initiated with antithyroid drugs, beta-blockers, glucocorticoids, and plasma exchange. Three days later, she developed focal neurological symptoms, and imaging studies revealed multiple ischemic lesions in the left middle cerebral artery territory. Further investigation confirmed left internal CAD, with no evidence of trauma or other underlying conditions to explain the dissection, leading to the conclusion that GD was the likely cause. The patient underwent thyroidectomy with subsequent clinical improvement, and she was discharged in good condition with long-term anticoagulation due to the presence of AF. This case highlights a rare association between GD and CAD, emphasizing the importance of considering CAD in patients with thyrotoxicosis who present with unexplained focal neurological symptoms. Early recognition and management of TS and CAD can improve clinical outcomes.

## Introduction

The dissection of cervical arteries is a frequent cause of ischemic stroke (IS) among young adults [1]. Likewise, hyperthyroidism can cause IS due to its association with atrial fibrillation (AF) [2], and it is a known risk factor for IS due to mechanisms not associated with AF [3]. Nevertheless, the coexistence of thyroid storm (TS) and IS is a rare presentation [2], and the association between carotid artery dissection (CAD) and thyrotoxicosis is even more so [4-6]. Here, we present a case of a 45-year-old woman with TS due to Graves' disease (GD) who presented with an IS due to left CAD.

This case was previously presented as a meeting abstract at the Asociación Colombiana de Endocrinología Endo Congress on May 10, 2023.

## Case presentation

A 45-year-old woman with an 18-month history of GD, late-onset diabetes mellitus, and arterial hypertension presented to the emergency department with a history of fever, vomiting, loss of appetite, abundant diarrhea, palpitations, fine tremors of the extremities, and asthenia for the past four days. Six months before the event, she had been hospitalized for an episode of TS. She lost 45 kg in the previous year and stopped going to work. Before admission, she was taking 60 mg of methimazole (1.2 mg/kg/day) and 100 mg of metoprolol daily, with no clinical control of symptoms or biochemical improvement. She had a previous Tc99 scintigraphy that showed goiter with increased diffuse uptake of 89%.

At physical examination, her blood pressure was 140/90 mmHg, heart rate was 130 beats/minute, and axillary temperature was 37.8°C. She had bilateral exophthalmos, a grade III painless goiter, and arrhythmic heart sounds with a pulse deficit. The rest of the physical exam was unremarkable.

The electrocardiogram evidenced AF, and the thyroid panel revealed a suppressed thyroid-stimulating hormone (0 milli-international units per milliliter; normal range: 0.55-4.78), with an elevated free T4 (5.32 ng/dL; normal range: 0.89-1.76). Other laboratory results are found in Table 1. The echocardiogram showed an enlargement of the left atrium (area of 19 cm^2^), and her Congestive heart failure, Hypertension, Age ≥75 years (2 points), Diabetes mellitus, prior stroke/transient ischemic attack/arterial thromboembolism (2 points), Vascular disease, age 65-74 years score (CHA2DS2-VASc) was 2 points (1 point for hypertension and 1 point for diabetes mellitus).

**Table 1:** Laboratory results INR: international normalized ratio; TSH: thyroid-stimulating hormone; HbA1C: hemoglobin A1c; LDL: low-density lipoprotein; IgM: immunoglobulin M; MPL: IgM phospholipid; IgG: immunoglobulin G; GPL: IgG phospholipid

Test	Result	Reference range
Hemoglobin (g/dL)	10.7	12-16
Hematocrit (%)	31.3	36-47
Leukocytes (cells/mm^3^)	4,800	4,500-11,000
Platelets (cells/mm^3^)	182,000	150,000-450,000
Serum creatinine (mg/dL)	0.24	0.57-1.11
Blood urea nitrogen (mg/dL)	6	9-23
Serum sodium (mmol/L)	143	136-145
Serum chloride (mmol/L)	106	98-107
Serum potassium (mmol/L)	2.9	3.5-5.1
Serum magnesium (mg/dL)	1.4	1.6-2.6
Serum calcium (mg/dL)	8.3	8.3-10.6
Serum phosphorus (mg/dL)	2.8	2.4-5.1
Aspartate aminotransferase (U/L)	11	9-52
Alanine aminotransferase (U/L)	16	14-36
Total bilirubin (mg/dL)	0.52	-
Direct bilirubin (mg/dL)	0.23	0-0.3
Albumin (g/dL)	3.7	3.5-4.5
Prothrombin time (seconds)	13.8	Control: 11.9
INR	1.23	-
Partial thromboplastin time (seconds)	34.6	Control: 29.4
Fibrinogen (mg/dL)	353	213-422
TSH (mUI/mL)	0	0.55-4.78
Free thyroxine (ng/dL)	5.32	0.89-1.76
HbA1C (%)	7.1	<5.7
Total cholesterol (mg/dL)	66	<200
LDL cholesterol (mg/dL)	22	Individualized
Triglycerides (mg/dL)	97	<150
IgM anticardiolipin (MPL; UmL)	5	0-6.9
IgG anticardiolipin (GPL)	3.5	0-9.9
IgM β2 glycoprotein (UA/mL)	3	Less than 10
IgG β2 glycoprotein (UA/mL)	2	Less than 10
Lupus anticoagulant	Negative	Negative

 The Burch-Wartofsky score was 50 points (temperature 10 points, gastrointestinal 10 points, heart rate 20 points, and AF 10 points), indicating a high TS probability. The Akamizu scale has been classified as TS1, which denotes an alternate combination of clinical manifestations, including thyrotoxicosis, tachycardia, fever, and gastrointestinal symptoms. The treatment was adjusted to methimazole, propranolol, glucocorticoids, and cholestyramine, and plasma exchange therapy was started as a bridging to thyroidectomy.

**Figure 1:**
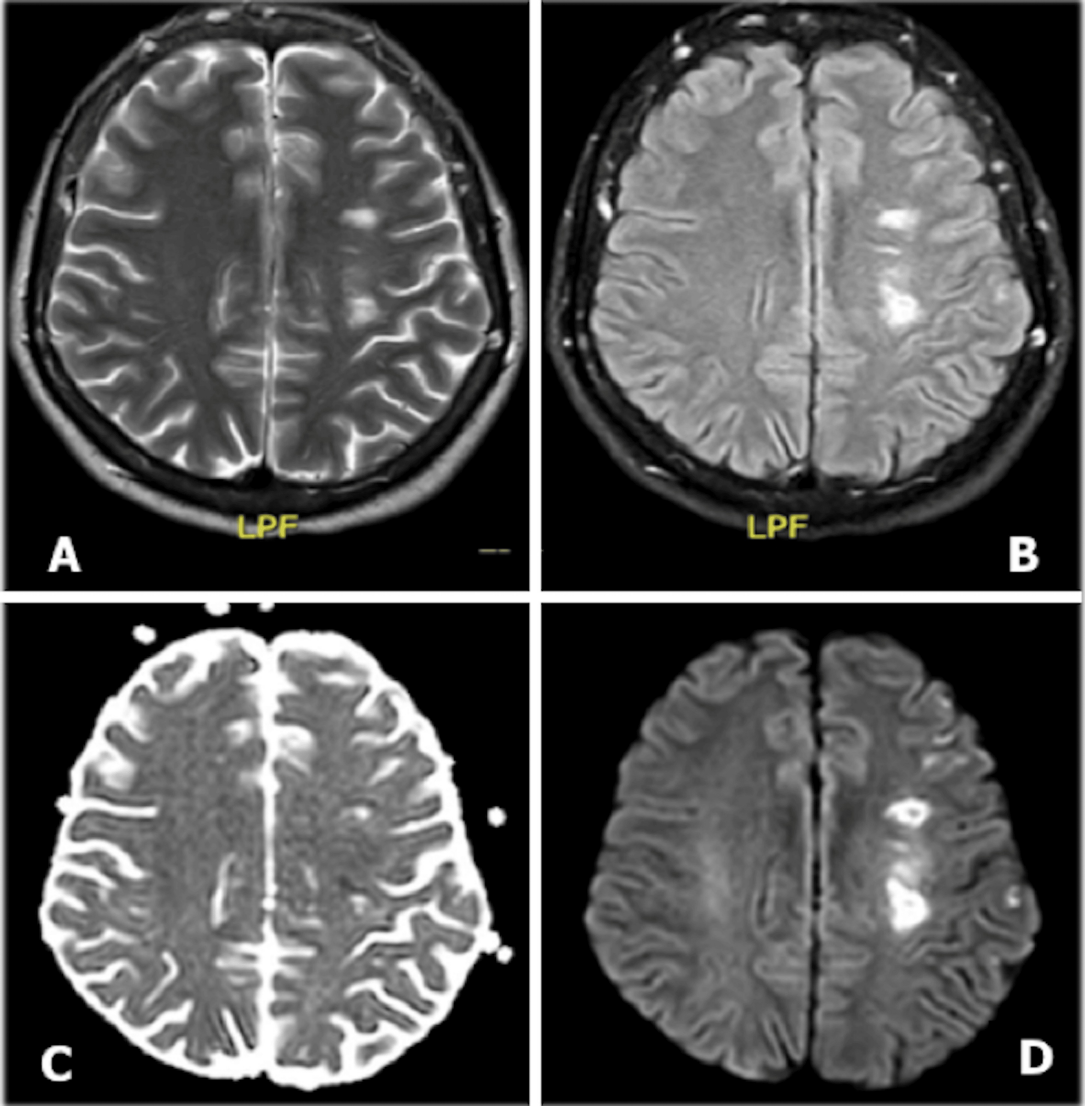
Magnetic resonance imaging with contrast of the brain. Ischemic infarcts in the middle cerebral artery territories, in different stages (acute, subacute, and chronic, with alteration of flow signal in the left carotid artery. (A) T2 sequence. (B) FLAIR sequence. (C) ADC sequence. (D) DWI sequence LPF: local perturbation field; FLAIR: fluid-attenuated inversion recovery; ADC: apparent diffusion coefficient; DWI: diffusion-weighted imaging

**Figure 2:**
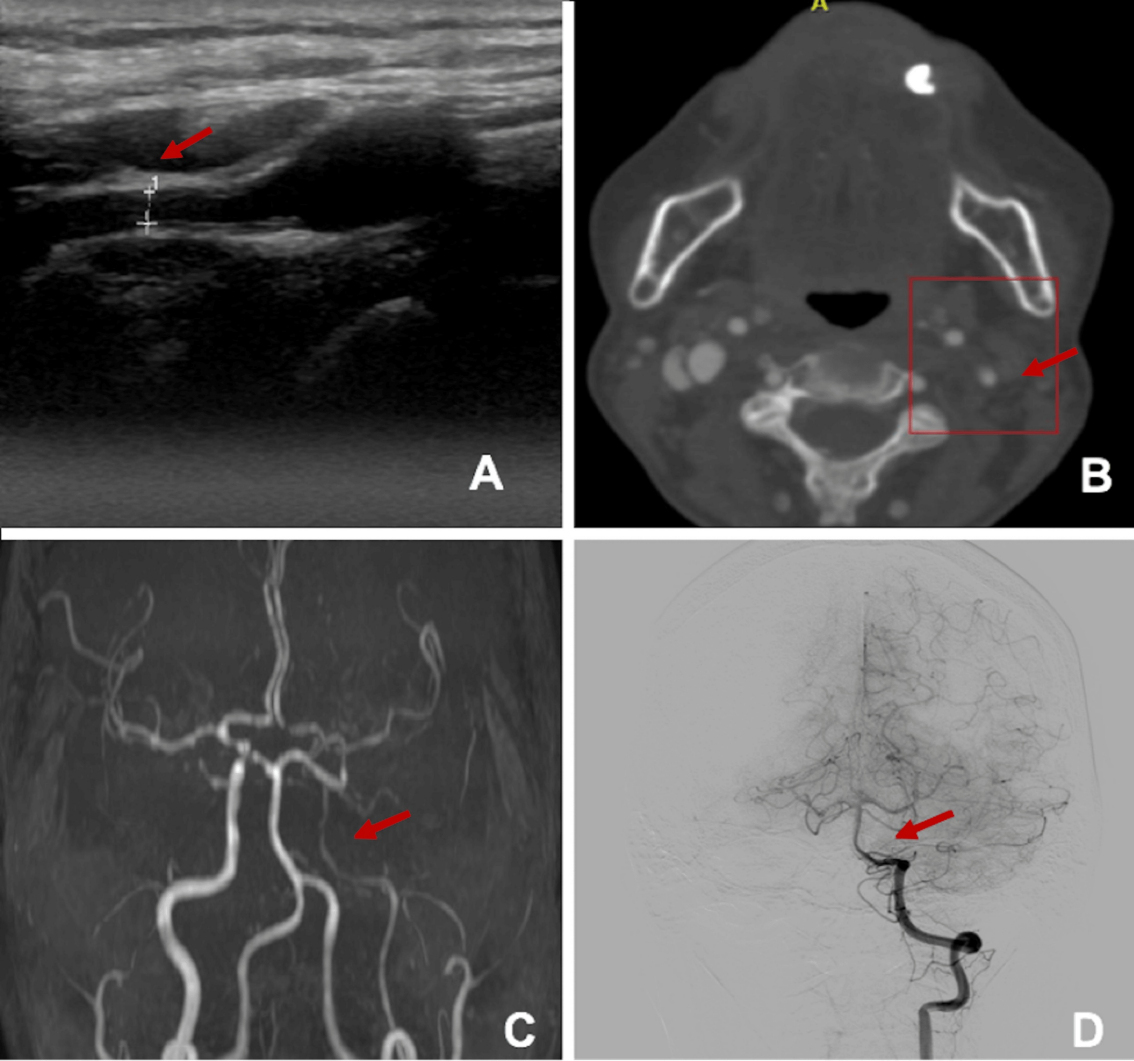
Reduction in the left internal carotid artery caliber in its cervical segment in (A) carotid Doppler ultrasonography and (B) neck CT angiography (red arrow), and with filiform discharge distal in its supraclinoid segments in (C) cranial MR angiography and (D) cerebral pan-angiography (red arrow) MR: magnetic resonance

Three days after admission, she presented with multiple focal motor crises in the right superior extremity, postictal paresis of the extremity, and dysarthria. The electroencephalogram evidenced multiple epileptiform discharges in the left posterior quadrant, and the cerebral magnetic resonance showed multiple infarcts secondary to hypoperfusion in the left middle cerebral artery territory in different states (Figure 1). A left internal carotid artery (ICA) diameter reduction was detected in the neck Doppler ultrasound, with evidence of intracranial extension in the neck CT angiography and cerebral pan-angiography (Figure 2).

Other possible causes were investigated. The patient did not smoke and had no history of head and neck trauma. Fibromuscular dysplasia was ruled out with a CT angiography of the neck, chest, and abdomen, and the antiphospholipid syndrome was not detected. She had no clinical features of autoimmune diseases other than GD or LADA. Since no other etiology could be found for the CAD, GD was determined as the underlying cause. Levetiracetam was started, and she had no other seizures. There was no indication to intervene in CAD. Due to the concurrent presence of AF with left atrium structural changes, long-term anticoagulation was initiated (CHA2DS2 -VA is now 4 points due to the stroke).

**Figure 3:**
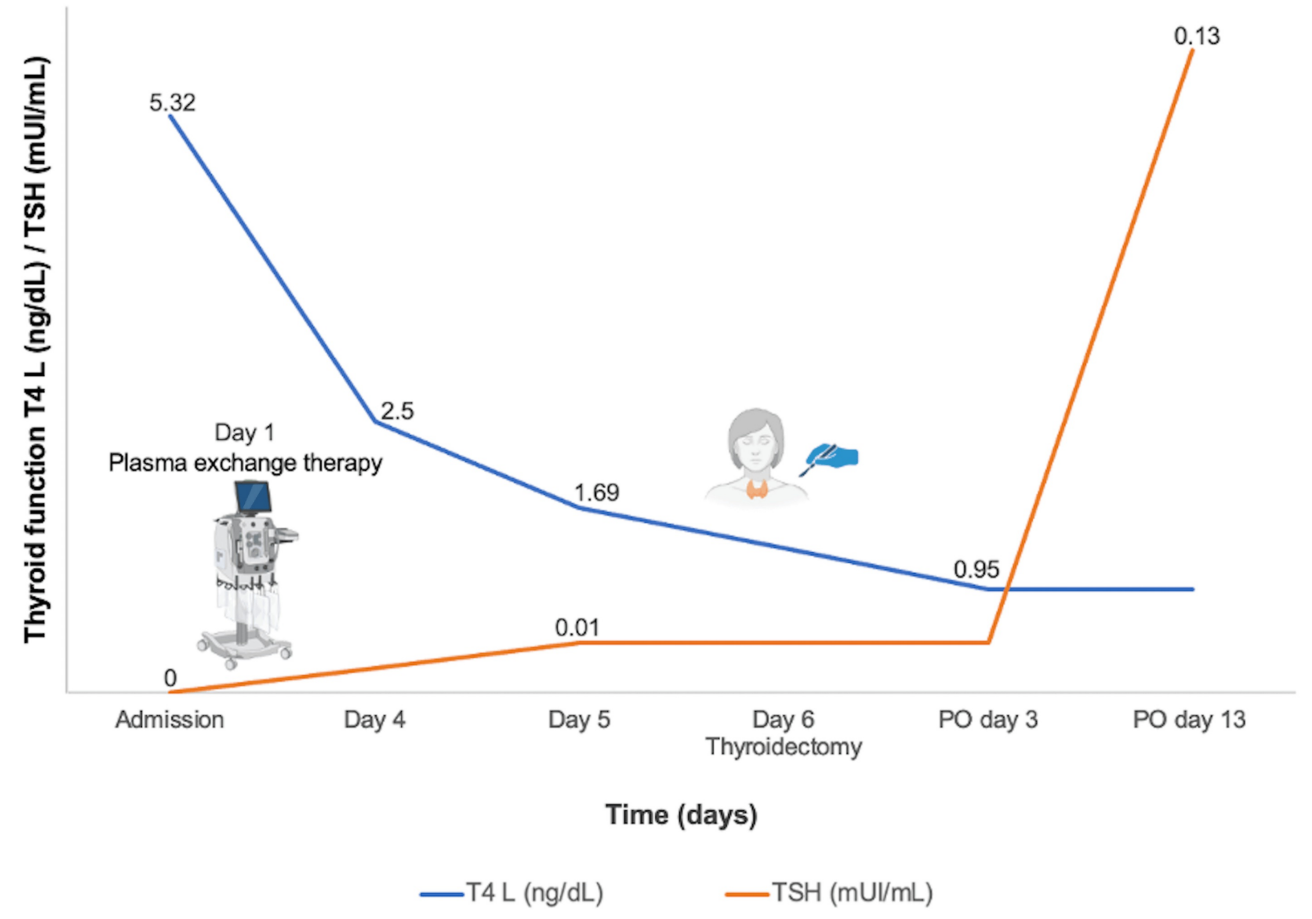
Changes in thyroid function over time TSH: thyroid-stimulating hormone; T4: thyroxine; PO: postoperative The image was created using BioRender

Thyroidectomy was performed during the sixth day of her hospital stay. Clinical improvement and progressive reduction of free T4 were achieved with said intervention (Figure 3). She presented with postsurgical hypoparathyroidism, which was controlled. Levothyroxine was started on postoperative day 5, and she was discharged on day 10 in good condition. A month after her discharge, she attended an endocrinology follow-up with full resolution of thyrotoxicosis symptoms.

## Discussion

Cervical artery dissection is a frequent cause of IS in young adults, accounting for 15%-20% of these cases [1]. The ICA is the most commonly affected vessel [7]. Causes and risk factors can be divided into traumatic (40%) and atraumatic (spontaneous dissection in 50% of cases). Traumatic dissections often result from high-energy accidents or minor incidents that precipitate dissection in an already compromised arterial wall. Predisposing factors for atraumatic dissection include smoking, arterial hypertension, connective tissue disorders, infections, styloid process elongation, fibromuscular dysplasia, migraine, and reversible cerebral vasoconstriction syndrome [8].

On the other hand, hyperthyroidism is a common condition (global prevalence of 0.2%-1.3%), and its most frequent etiology is GD, which predominantly affects young adults [9]. Hyperthyroidism may cause IS due to its relationship with AF, which occurs in 10%-35% of cases [2]. However, IS may also occur through non-AF-mediated mechanisms, including hypercoagulability, hypofibrinolysis, endothelial dysfunction, arterial hypertension, increased intimal stiffness and thickness, and accelerated atherosclerosis [2,10-14], leading to cerebral venous thrombosis, and stenosis of intracranial and carotid arteries [5,15]. The coexistence of TS and IS is rare, with the latter potentially being a direct consequence or a precipitating factor [2].

Moyamoya disease has been associated with GD. It is characterized by the occlusion of the terminal portion of the bilateral ICAs, with the formation of collateral vessels in the form of a web in the cerebral base, leading to IS or cerebral hemorrhage [16]. In GD, vascular compression due to goiter has also been described, as well as antiphospholipid syndrome and giant cell arteritis [3,17].

The association between CAD and thyrotoxicosis is very rare, with few reported cases. Campos et al. reported two cases [4], Winter et al. documented one case [5], and Choi et al. described another case [6], all of which presented with bilateral dissection in the context of GD. Here, we describe the case of a patient with GD who had a unilateral CAD. To our knowledge, this is the first case of GD with unliteral CAD reported in the literature.

The mechanisms underlying this association are not entirely clear, but thyroid autoimmunity plays a crucial role, contributing to vascular wall damage through inflammation [5]. In a case-control study of 58 patients with IS, a greater prevalence of thyroid autoimmunity was identified in those with cervical artery dissection (31% vs. 6.9%, p = 0.041) [18]. Similarly, in another study involving 215 cases of craniocervical artery dissections and 430 controls, thyroid autoimmunity was identified in 7% of cases and in none of the controls (p < 0.001) [19]. Notably, all reported cases [4-6] and our case involved patients with GD. We did not find any case reports of this entity due to nonautoimmune causes of thyrotoxicosis.

The recommended treatment includes initiating anticoagulation or antiplatelet therapy alongside managing cardiovascular risk factors and the underlying etiology. Surgical or endovascular interventions are reserved for refractory cases [20]. In the case of our patient, long-term anticoagulation was initiated due to the presence of AF and left atrial enlargement.

## Conclusions

An apparent association exists between GD and CD; however, further research is needed to confirm this relationship. Investigating this link will enhance our understanding of the mechanisms-beyond AF and a hypercoagulable state-through which hyperthyroidism may predispose patients to IS.

We recommend that emergency medicine and critical care physicians consider CAD in patients with GD who present with focal neurological symptoms, symptoms not typically associated with thyrotoxicosis, or symptoms that are otherwise unexplained. We also suggest the evaluation of thyroid function in young adults presenting with IS of unknown cause or spontaneous CAD of unclear etiology.
